# Impact of early life geohelminths on wheeze, asthma and atopy in Ecuadorian children at 8 years

**DOI:** 10.1111/all.14821

**Published:** 2021-04-07

**Authors:** Philip J. Cooper, Irina Chis Ster, Martha E. Chico, Maritza Vaca, Yisela Oviedo, Augusto Maldonado, Mauricio L. Barreto, Thomas A. E. Platts‐Mills, David P. Strachan

**Affiliations:** ^1^ Institute of Infection and Immunity St George's University of London London UK; ^2^ School of Medicine Universidad Internacional del Ecuador Quito Ecuador; ^3^ Fundacion Ecuatoriana Para La Investigacion en Salud Quito Ecuador; ^4^ Colegio de Ciencias de la Salud Universidad San Francisco de Quito Quito Ecuador; ^5^ CIDACS‐FIOCRUZ Salvador Brazil; ^6^ Division of Allergy and Clinical Immunology University of Virginia Charlottesville VA USA; ^7^ Population Health Research Institute St George's University of London London UK

**Keywords:** asthma, atopy, Ecuador, geohelminths, wheeze

## Abstract

**Background:**

Early‐life exposures to geohelminths may protect against development of wheeze/asthma and atopy.

**Objective:**

To study the effect of maternal geohelminths and infections in children during the first 5 years on atopy, wheeze/asthma and airways reactivity/inflammation at 8 years.

**Methods:**

Birth cohort of 2404 neonates followed to 8 years in rural Ecuador. Data on wheeze/asthma were collected by questionnaire and atopy by skin prick test (SPT) reactivity to 9 allergens. We measured airways reactivity to bronchodilator, fractional exhaled nitric oxide (FeNO) and nasal eosinophilia. Stool samples were examined for geohelminths by microscopy.

**Results:**

1933 (80.4%) children were evaluated at 8 years. Geohelminths were detected in 45.8% of mothers and 45.5% of children to 5 years. Frequencies of outcomes at 8 years were as follows: wheeze (6.6%), asthma between 5 and 8 years (7.9%), SPT (14.7%), airways reactivity (10%) and elevated FeNO (10.3%) and nasal eosinophilia (9.2%). Any maternal geohelminth was associated with reduced SPT prevalence (OR 0.72). Childhood *Trichuris trichiura* infections during the first 5 years were associated with reduced wheeze (OR 0.57) but greater parasite burdens with *Ascaris lumbricoides* at 5 years were associated with increased wheeze (OR 2.83) and asthma (OR 2.60). Associations between maternal geohelminths and wheeze/asthma were modified by atopy. Parasite‐specific effects on wheeze/asthma and airways reactivity and inflammation were observed in non‐atopic children.

**Conclusions:**

Our data provide novel evidence for persistent effects of *in utero* geohelminth exposures on childhood atopy but highlight the complex nature of the relationship between geohelminths and the airways. Registered as an observational study (ISRCTN41239086).

## Graphical abstract

Maternal geohelminths protect 8‐year‐old children from the development of allergen skin prick test reactivity with strongest effects observed in infected children of infected mothers. Early childhood infections with *Trichuris trichiura* protect against wheeze at 8 years. Effects of geohelminths on wheeze/asthma and airways reactivity and inflammation at 8 years vary by parasite species and atopy.

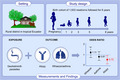

**Abbreviations**
Adj.: adjusted
CD4+: Cluster of differentiation 4
CI: confidence interval
Epg: eggs per gramme
FeNO: fractional exhaled nitric oxide
FEV_1_
: forced expiratory volume in 1 second
LMIC: low‐ and middle‐income country
OR: Odds ratio
SES: socio‐economic status
Spp.: species
SPT: allergen skin prick test
Th: T helper cell type
Vs.: versus

## 1 INTRODUCTION

Asthma is the most common chronic disease of childhood and is estimated to affect 358 million worldwide.^1^ Asthma is increasing in prevalence in many low‐ and middle‐income countries (LMICs).^2^ Temporal trends of increasing asthma prevalence in LMICs are considered to be related to urbanization and loss of protective exposures associated with rural residence.^3^

Recent years have seen increasing urbanization in LMICs, accompanied by reductions in poverty, improved access to basic services and transformation of the living environment.^3^ Under such circumstances, the intensity of microbial exposures in early childhood is likely to have declined, affecting the maturation and regulation of the immune system and risk of inflammatory diseases including asthma.^4^, ^5^

Geohelminths (caused by *Ascaris lumbricoides*, *Trichuris trichiura* and hookworm) infect over 1 billion human worldwide^6^ and are most prevalent among children living in conditions of poverty in tropical regions of LMICs. The most frequent geohelminths found in coastal Ecuador are *Ascaris* (*A. lumbricoides*) and *Trichuris* (*T. trichiura)* ^7^ that cause significant morbidity, particularly in children, through their effects on nutritional status, growth and cognition.^8^ Geohelminths cause chronic infections that are associated with modulation of host Th2 inflammatory mechanisms.^8^ The tight regulation of Th2 inflammatory responses may modulate inflammation associated with allergy and asthma. It has been suggested that the lower prevalence of asthma symptoms observed in rural compared to urban populations in tropical regions of LMICs^3^ is explained by the immune modulatory effects of endemic geohelminth infections.^5^

Epidemiological studies of the relationship between geohelminths and atopy or asthma have shown conflicting findings in cross‐sectional analyses and intervention studies done largely in schoolchildren.^9^, ^10^, ^11^, ^12^, ^13^, ^14^, ^15^ We hypothesized that *in utero* or early childhood exposures to geohelminths protect against the development of atopy and asthma in later childhood. To test this hypothesis, we followed an Ecuadorian cohort from birth to 8 years of age in an area of high endemicity. Previous analyses from the cohort showed a protective effect of maternal geohelminths on atopy to mite allergens at 3 years.^16^ At 5 years, we observed that maternal infections increased the risk of wheeze while childhood infections protected against wheeze and atopy to perennial aeroallergens and raised the possibility that childhood infections might modulate wheeze through non‐allergic mechanisms.^17^

To understand better the effects of early childhood geohelminth exposures on wheeze, asthma and atopy, and whether the previously observed effects persist, we report findings at 8 years including novel measurements of airways inflammation and reactivity to provide further insights on effects of geohelminths on non‐atopic wheezing illness and asthma.

## 2 METHODS

### 2.1 Study design, setting and participants

A prospective study from birth was done in the District of Quininde in Esmeraldas Province, Ecuador, as described.^18^ The District serves a population of approximately 150,000 with limited access to basic services. The District is largely rural with economic activities based mainly on agriculture. The District includes 3 towns of 10,000 or more inhabitants that contain within municipal urban boundaries, rapidly expanding peri‐urban populations representing the poorer segment of the population living in precarious circumstances with limited access to basic services. Neonates were recruited at a public hospital between November 2005 and December 2009. Follow‐up evaluations and sample collections were done at 13 months and at 2, 3, 5 and 8 years of age.

### 2.2 Study procedures

A questionnaire was used to collect data on sociodemographic factors, family history of allergy, and home environment by interview of the child's mother around the time of birth. Questionnaires were repeated periodically for wheeze and asthma symptoms. Wheeze was defined as any episode of wheeze during the previous 12 months at 8 years. Asthma was defined as wheeze during the previous 3 years plus one or both of parentally reported wheeze up to 5 years and a doctor diagnosis of asthma ever.

Stool samples to detect geohelminths were collected from mothers before birth and from children periodically from birth. Samples were examined using a combination of saline mounts, modified Kato‐Katz, formol‐ether concentration, and carbon‐coproculture methods.^19^ A positive sample was defined by the presence of at least one egg or larva from any of the four detection methods. *Ascaris* and *Trichuris* infection intensities were expressed as eggs per gram (epg) of faeces.

Spirometry was done at 8 years using a MicroLoop spirometer (CareFusion, UK) before and after 200 μg salbutamol administered. A positive test for airways reactivity was an increase in FEV_1_ of ≥12%. Fractional exhaled nitric oxide was measured in parts per billion using NObreath (Bedfont Scientific, UK). Nasal wash samples were collected at 8 years as described.^20^

Atopy was measured by SPTs with 9 allergen extracts (Greer laboratories, Lenoir, North Carolina, USA): house dust mites (*Dermatophagoides pteronyssinus*/*Dermatophagoides farinae* mix), American cockroach, cat, dog, grass pollen (9 southern grass mix), fungi (New stock mix), egg, milk, and peanut, with positive histamine and negative saline controls. A positive reaction was defined as a mean wheal diameter at least 3 mm greater than the saline control 15 min after pricking the allergen onto the forearm with lancets. Positive SPT was defined as a positive reaction to any of the allergens.

### 2.3 Statistical analysis

To measure effects of geohelminths on asthma prevalence with >80% power at significance level of 0.05, we estimated that we would need to follow up 1725 children to detect a difference in asthma prevalence of ≥6% with infection risks of 50% among mothers and 35% among children. Primary exposures were maternal and childhood geohelminth infections, and primary outcomes were wheeze, asthma, and SPT to any allergen. Exploratory analyses addressed the effects of geohelminth species and infection intensities on primary outcomes, and effects of geohelminths on airways reactivity and airways inflammation. Univariable and multivariable logistic regression were used to estimate associations. Potential confounders are shown in Table 1. Urban‐rural residence was defined by municipal geographic boundaries. A socio‐economic status (SES) index was created using principal component analysis of 7 socio‐economic variables.^16^ A conservative analytic approach was used for all adjusted analyses in which potential confounders included were those with *p* < .05 in univariable analyses for any of the primary outcomes. All statistical analyses were done using Stata 11 (StataCorp, College Station, Tex).

**TABLE 1:** Frequencies of maternal and childhood geohelminth infections to 5 years of age and potential confounders and associations with wheeze and allergen skin test (SPT) reactivity to any allergen at 8 years and asthma between 5 and 8 years

Variable	Overall	Wheeze			Asthma			SPT to any allergen		
	*n* (%)	%	OR (95% CI)	*p* value	%	OR (95% CI)	*p* value	%	OR (95% CI)	*p* value
Any maternal geohelminth										
No	1048 (54.2)	6.2	1		8.2	1		16.8	1	
Yes	885 (45.8)	7.0	1.14 (0.79–1.63)	.478	7.6	0.92 (0.66–1.28)	.606	12.2	**0.69 (0.53–0.89)**	**.005**
Any childhood geohelminths										
No	1054 (54.5)	6.7	1		8.4	1		16.4	1	
Yes	879 (45.5)	6.4	0.94 (0.66–1.35)	.747	7.4	0.88 (0.63–1.22)	.439	13.0	**0.75 (0.59–0.98)**	**.034**
Maternal age (years)										
≤20	501 (25.9)	5.8	1		6.6	1		13.8	1	
21–29	929 (48.1)	6.6	1.14 (0.72–1.80)	.564	8.7	1.35 (0.89–2.06)	.157	14.0	1.01 (0.74–1.40)	.908
≥30	503 (26.0)	7.4	1.29 (0.78–2.14)	.317	7.8	1.19 (0.74–1.93	.474	16.9	1.27 (0.90–1.80)	.170
Maternal ethnicity										
Afro‐Ecuadorian	508 (26.3)	7.7	1		11.8	1		14.8	1	
Non‐Afro‐Ecuadorian	1425 (73.7)	6.2	0.79 (0.54–1.17)	.242	6.5	**0.52 (0.37–0.73)**	**<.001**	14.7	0.99 (0.75–1.32)	.958
Maternal educational level										
Illiterate	293 (15.2)	5.5	1		8.2	1		14.7	1	
Complete primary	1133 (58.6)	6.2	1.14 (0.65–1.99)	.646	7.3	0.89 (0.55–1.42)	.616	13.7	0.92 (0.64–1.33)	.661
Complete Secondary	507 (26.2)	8.2	1.52 (0.84–2.77)	.167	9.1	1.12 (0.67–1.87)	.671	17.0	1.19 (0.89–1.77)	.397
Area of residence										
Urban	1346 (69.6)	7.4	1		9.4	1		15.5	1	
Rural	587 (30.4)	4.6	**0.60 (0.39–0.93)**	**.022**	4.6	**0.47 (0.30–0.72)**	**<.001**	13.0	0.81 (0.61–1.08)	.153
Sex										
Male	984 (50.9)	7.9	1		8.7	1		16.5	1	
Female	949 (49.1)	5.2	**0.63 (0.44–0.91)**	**.015**	7.1	0.79 (0.57–1.11)	.172	12.9	**0.75 (0.58–0.96)**	**.025**
Socio‐economic status^a^										
1	642 (33.2)	6.2	1		7.8	1		13.4	1	
2	638 (33.0)	5.6	0.90 (0.57–1.43)	.656	7.2	0.92 (0.61–1.40)	.695	14.0	1.05 (0.76–1.44)	.773
3	653 (33.8)	7.8	1.28 (0.83–1.96)	.267	8.7	1.13 (0.76–1.68)	.539	16.7	1.30 (0.95–1.76)	.098
Birth order										
1st	490 (25.4)	6.1	1		5.5	1		16.7	1	
2nd−4th	1063 (55.0)	7.3	1.21 (0.79–1.88)	.382	9.4	**1.78 (1.15–2.76)**	**.010**	13.5	1.05 (0.76–1.44)	.088
≥5th	380 (19.6)	5.0	0.81 (0.45–1.46)	.477	6.8	1.26 (0.72–2.20)	**.416**	15.5	1.30 (0.95–1.76)	.632
Maternal allergy										
No	1830 (95.4)	6.3	1		7.7	1		14.4	1	
Yes	89 (4.6)	12.4	**2.10 (1.09–4.06)**	**.027**	14.6	**2.06 (1.12–3.81)**	**.020**	18.0	1.30 (0.75–2.27)	.355
Household overcrowding^b^										
≤3	1086 (56.2)	6.5	1		7.6	1		15.6	1	
>3	847 (43.8)	6.6	1.01 (0.70–1.45)	.948	8.4	1.12 (0.80–1.56)	.502	13.6	0.85 (0.66–1.10)	.222
Pets inside house										
No	1438 (74.4)	6.3	1		7.7	1		14.5	1	
Yes	495 (25.6)	7.3	1.16 (0.78–1.73)	.465	8.7	1.15 (0.79–1.66)	.461	15.2	1.05 (0.79–1.40)	.738
Large farm animals^c^										
No	1294 (66.9)	7.0	1		8.0	1		15.8	1	
Yes	639 (33.1)	5.8	0.82 (0.55–1.22)	.331	7.7	0.95 (0.67–1.35)	.778	12.5	0.76 (0.58–1.01)	.058
Pneumonia to 13 months										
No	1758 (95.3)	6.6	1		7.9	1		15.0	1	
Yes	90 (4.7)	5.6	0.83 (0.33–2.09)	.697	7.8	0.99 (0.45–2.18)	.980	7.8	0.48 (0.22–1.04)	.064

Note

SPT—allergen skin prick test reactivity to any of 9 allergens. Odds ratios (OR) and 95% confidence intervals (95% CI) for univariable associations were estimated using logistic regression. *p* < .05 are shown in bold. Ethnicity ‘other’ represents: 1417 Mestizo/8 Indigenous. Numbers of missing values (brackets) were as follows: maternal allergy (14) and pneumonia during first 13 months (85). Other helminths: mother (S. stercoralis, 4.0%; Hymenolepis spp., 0.5%); child (hookworm, 1.1%; S.stercoralis, 1.5%; Hymenolepis spp., 4.2%).

^a^ Socio‐economic status (SES) represents tertiles of *z* scores obtained using a factor analysis with 1 representing the lowest and 3 the highest SES.

^b^ Household overcrowding is defined as the number of people living in the household per sleeping room.

^c^ Any of cows, pigs, mules, donkeys and horses.

### 2.4 Ethical considerations

Study protocols were approved by ethics committees in Ecuador (Hospital Pedro Vicente Maldonado, Universidad San Francisco de Quito, and Universidad Internacional del Ecuador) and UK (London School of Hygiene and Tropical Medicine). The study is registered as an observational study (ISRCTN41239086). Informed written consent was obtained from the child's mother, and minor assent was obtained from the child at 8 years. Anthelmintic treatment was provided to mothers and children with positive stools for geohelminths as recommended.^21^

## 3 RESULTS

### 3.1 Cohort participants

**FIGURE 1:**
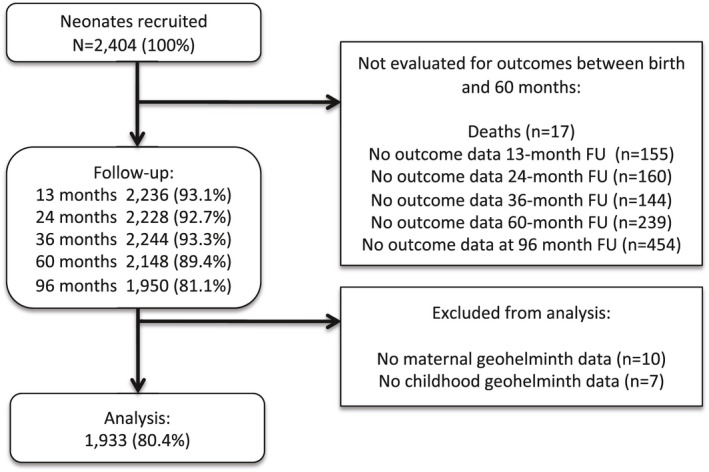
Participant flow through follow‐up to 8 years of age and those included and excluded from the analysis. FU, follow‐up

Analyses at 8 years of age were done using data from 1933 (80.4%) children of 2404 newborns initially recruited and for whom complete data were available on primary exposures and outcomes (Figure 1). Frequencies of potential confounders for children included in and excluded from the analysis were similar (Table S1).

### 3.2 Frequencies of exposures and outcomes

Almost half (45.8%) the children had an infected mother (*Ascaris* 27.6%, *Trichuris* 28.9%, hookworm 5.6% and *Strongyloides stercoralis* 4.0%). Geohelminth infections during the first 5 years were observed in 45.5% of 1933 children analysed at 8 years, most frequently with *Ascaris* (36.3%) and *Trichuris* (25.5%). Other infections were hookworm (1.1%), *S. stercoralis* (1.5%) and *Hymenolepis* spp. (4.2%). Geometric mean infection intensities at 5 years among infected children were 1162 epg for *Ascaris* and 227 epg for *Trichuris*. Maternal and childhood geohelminth infections were strongly associated; of 1933 children, 35.5% had neither maternal nor childhood infections, 19.0% had maternal geohelminths only, 18.7% had childhood infections only, and 26.8% had both (*p* < .001). At least one episode of wheeze from birth to 8 years of age was reported for 38.0% of children. Wheeze prevalence at 8 years was 6.6%, and asthma between 5 and 8 years was 7.9%. SPT prevalence at 8 years was 14.7%: *D. pteronyssinus*/*farinae* 10.7%, cockroach 5.3%, mixed fungi 0.3%, dog 0.1%, cat 0.2%, mixed grasses 1.1%, peanut 0.3%, milk 0.1% and egg 0.1%. Airways reactivity, elevated FeNO, (>35 ppb) and nasal eosinophilia (>5%) were observed in 10%, 10.3% and 9.2%, respectively, of children.

### 3.3 Maternal geohelminth protect against atopy

**FIGURE 2:**
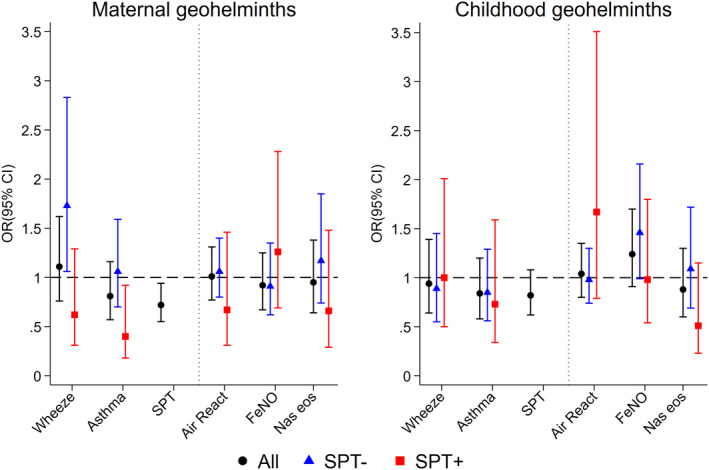
Adjusted associations between maternal and childhood geohelminths and study outcomes among all children and stratified by atopy (SPT). Shown are adjusted ORs and 95% CIs. Air React., airways reactivity; FeNO, fractional exhaled nitric oxide; Nas eosin, nasal eosinophilia; SPT, allergen skin prick test reactivity

Before adjustment for potential non‐helminth confounders, both maternal geohelminths and childhood geohelmiths were associated with a significant reduction in SPT positivity, but not wheeze or asthma (Table 1). The univariate association of maternal helminths with atopy (OR 0.69) was not attenuated by adjustment for non‐helminth confounders (OR 0.68, 95% CI 0.52–0.89, *p* = .004) and remained little changed by further adjustment for childhood geohelminths (OR 0.72, Table 2 and Figure 2). The unadjusted association of childhood geohelminths with atopy was of similar magnitude (OR 0.75) and was barely altered by adjustment for non‐helminth confounders (OR 0.77, 95% CI 0.59–1.01, *p* = .054) but became somewhat weaker with further adjusted for maternal helminths (OR 0.82, Table 2 and Figure 2).

**TABLE 2:** Adjusted analyses for associations between maternal and childhood geohelminth infections to 5 years of age or potential confounders and associations with wheeze and allergen skin prick test (SPT) reactivity to any allergen at 8 years and asthma between 5 and 8 years

Variable	Wheeze		Asthma		SPT to any allergen	
	OR (95% CI)	*p* value	OR (95% CI)	*p* value	OR (95% CI)	*p* value
Any maternal geohelminth						
No	1		1		1	
Yes	1.11 (0.76–1.62)	.594	0.81 (0.57–1.16)	.254	**0.72 (0.55–0.94)**	**.018**
Any childhood geohelminth						
No	1		1		1	
Yes	0.94 (0.64–1.39)	.740	0.84 (0.58–1.20)	.329	0.82 (0.62–1.08)	.157
Maternal allergy						
No	1		1		1	
Yes	**2.24 (1.14–4.39)**	**.019**	**2.07 (1.10–3.88)**	**.024**	1.37 (0.78–2.41)	.274
Maternal ethnicity						
Afro‐Ecuadorian	1		1		1	
Non‐Afro‐Ecuadorian	0.88 (0.58–1.34)	.551	**0.55 (0.38–0.79)**	**.001**	0.95 (0.70–1.28)	.725
Area of residence						
Urban	1		1		1	
Rural	**0.60 (0.38–0.97)**	**.035**	**0.46 (0.29–0.73)**	**.001**	0.84 (0.62–1.14)	.269
Sex						
Male	1		1		1	
Female	**0.60 (0.41–0.88)**	**.009**	0.78 (0.55–1.09)	.149	**0.70 (0.54–0.90)**	**.006**
Birth order						
1st	1		1		1	
2nd–4th	1.20 (0.77–1.87)	.416	**1.87 (1.19–2.92)**	**.006**	0.81 (0.60–1.09)	.168
≥5th	0.78 (0.43–1.45)	.440	1.26 (0.71–2.24)	.438	1.02 (0.70–1.48)	.930
Large farm animals‡						
No	1		1		1	
Yes	0.91 (0.60–1.38)	.650	1.17 (0.80–1.71)	.431	0.81 (0.60–1.09)	.171

Note

SPT—allergen skin prick test reactivity to any of 9 allergens. Odds ratios (OR) and 95% confidence intervals (95% CI) were estimated using logistic regression and adjusted for all variables shown. *p* < .05 are shown in bold.

‡ Any of cows, pigs, mules, donkeys and horses.

### 3.4 Childhood trichuriasis protects against wheeze but ascariasis increase risk of wheeze and asthma

**FIGURE 3:**
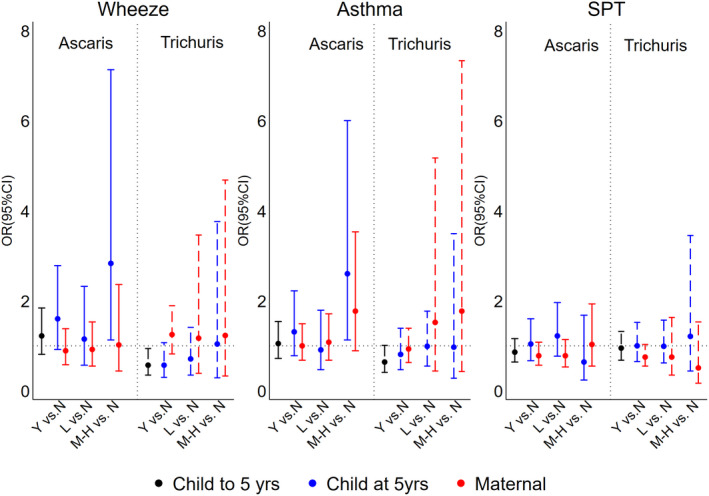
Adjusted associations between geohelminth parasite species and parasite burdens in mothers and children up to and at 5 years of age, and wheeze, asthma and atopy (SPT). Y = yes (infected); N = no (uninfected); L, light parasite burdens; M‐H, moderate to heavy parasite burdens. Shown are adjusted ORs and 95% CIs. SPT, allergen skin prick test reactivity

Geohelminth infections to 13 months, 2 and 3 years of age were not significantly associated with primary outcomes (Table S2). Maternal geohelminth parasite species or parasite burden were not associated with primary outcomes (Figure 3 and Table S3). The presence of any *Trichuris* infection within the first 5 years of life was associated with a reduced prevalence of wheeze (adj. OR 0.57, 95% CI 0.35–0.94, *p* = .029), while moderate to heavy parasite burdens with *Ascaris* at 5 years were associated with increased wheeze (adj. OR 2.83, 95% CI 1.13–7.13, *p* = .027) and asthma (adj. OR 2.60, 95% CI 1.13–6.00, *p* = .025) (Figure 3).

### 3.5 Strongest protective effects against SPT were seen among infected children of infected mothers

Chronic exposures to childhood geohelminth infections were assessed as repeated infections in childhood (i.e. 0, 1, and >=2 documented infections with *Ascaris* or *Trichuris* during the first 5 years of life), and as cumulative burdens for *Ascaris* or *Trichuris* during the first 5 years of life. None of these had significant effects on outcomes (data not shown). Maternal geohelminths were strongly associated with childhood infections (adj. OR 2.70, 95% CI 2.23–3.22, *p* < .001). To separate maternal/childhood geohelminth effects, we did a four‐group analysis of combinations of maternal and childhood geohelminth infections (mother−/child−, mother+/child−, mother−/child+, and mother+child+). Significant effects were observed on SPT for the mother+/child+group (versus mother‐/child‐, adj. OR 0.58, 95% CI 0.41–0.83, *p* = .003) (Table S4).

### 3.6 Children of mothers with greater ascariasis infection intensities have greater levels of FeNO

There were no significant associations of geohelminths with airways reactivity, FeNO and nasal eosinophilia (Figure 2 and Table S5). When considering parasite species and burden, elevated FeNO was associated with moderate/heavy parasite burdens with *Ascaris* both in mothers (vs. uninfected, adj. OR 2.19, 1.23–3.90, *p* = .008) and children at 5 years (vs. uninfected, adj. OR 2.27, 1.10–4.70, *p* = .027). After co‐adjusting for maternal and child infection intensities, only the maternal effect remained significant (vs. uninfected, adj. OR 2.20, 95% CI 1.16–4.19, *p* = .016).

### 3.7 SPT modifies association between maternal geohelminths and wheeze/asthma

SPT reactivity was strongly associated with wheeze (adj. 4.13, 95% CI 2.80–6.08, *p* < .001) and asthma (adj. OR 2.32, 95% CI 1.57–3.42, *p* < .001). We explored if effects of geohelminths on outcomes might vary by SPT (Figure 2 and Table S6). Although interactions were seen for SPT on geohelminth‐outcome associations, they were not highly significant. However, overall associations between maternal geohelminths and wheeze/asthma were negative among atopic but positive among non‐atopic children.

### 3.8 Maternal geohelminth parasite species are associated with childhood wheeze/asthma and airways reactivity and inflammation among non‐atopics

**FIGURE 4:**
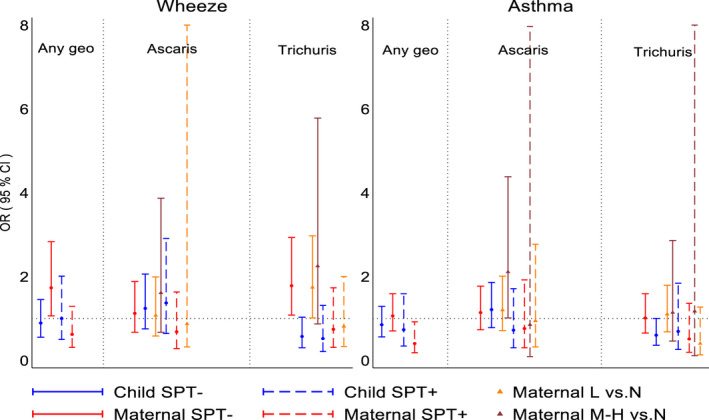
Adjusted associations between geohelminths, geohelminth parasite species and parasite burdens in mothers and children up to 5 years of age and wheeze/asthma stratified by atopy (SPT). L, light parasite burdens; M‐H, moderate to heavy parasite burdens. Shown are adjusted ORs and 95% CIs. SPT, allergen skin prick test reactivity

Among non‐atopic children, maternal geohelminths were positively associated with wheeze (adj. OR 1.73, 95% CI 1.06–2.83, *p* = .028), an effect that appeared to be explained by maternal *T. trichiura* infections (adj. OR 1.78, 95% CI 1.08–2.93, *p* = .024), while a maternal effect on asthma was associated with moderate to heavy infection intensities with *Ascaris* (vs. uninfected, adj. 2.11, 95% CI 1.01–4.38, *p* = .046) (Figure 4 and Table S6). To separate contrasting effects of maternal vs. childhood *Trichuris* on wheeze in non‐atopic children, we did a 4‐group analysis by strata of maternal/child *Trichuris* infection using maternal−/child− as reference group: we observed that mother+/child− children had an elevated risk of wheeze (adj. OR 2.39, 95% CI 1.39–4.10, *p* = .002), an effect that was abolished by childhood infections (mother+/child+, adj. OR 0.94, 0.42–2.07, *p* = .858) (Table S7). Neither any maternal nor any childhood geohelminth infections were associated with airways reactivity, elevated FeNO or nasal eosinophilia irrespective of atopy (Table S8). Analyses by parasite species and burden showed effects among non‐atopic children (Table S9): (1) light infection intensities with *Trichuris* in mothers were positively (vs. uninfected, adj. OR 1.56, 95% CI 1.05–2.01, *p* = .028) but childhood *Trichuris* infections inversely (adj. OR 0.62, 95% CI 0.40–0.96, *p* = .031) associated with airways reactivity; (2) childhood *Ascaris* (adj. OR 1.61, 95% CI 1.07–2.42, *p* = .021) and moderate/heavy infection intensities with *Ascaris* in mothers (vs. uninfected, adj. OR 2.89, 95% CI 1.53–5.49, *p* = .001) were positively associated with elevated FeNO; and (3) nasal eosinophilia was associated with moderate/heavy infections with *Ascaris* in mothers (vs. uninfected, adj. OR 2.27, 95% CI 1.00–5.12, *p* = .049).

## 4 DISCUSSION

We tested the hypothesis that early‐life exposures to geohelminths—through an infected mother during pregnancy or early childhood, or both—protect against wheeze/asthma and atopy at school age. To do this, we followed a birth cohort study to measure effects of maternal and early childhood geohelminths on the development of atopy (measured as SPT), wheeze/asthma and airways reactivity and inflammation(measured by elevated FeNO and nasal eosinophilia) at 8 years. Our findings indicate that maternal geohelminths have persistent protective effects against childhood SPT but that this effect was strongest among children of infected mothers who also acquired infections. A maternal effect on increased wheeze and airways inflammation was seen among non‐atopic children, the dominant phenotype in non‐affluent societies.^22^, ^23^ Effects on SPT were not associated with specific parasite species, while the maternal effect on wheeze among non‐atopic children appeared to be mediated by *Trichuris* infection. In contrast, early childhood *Trichuris* protected against wheeze.

There are few previous longitudinal analyses of the effects of early geohelminth infections on development of allergy, and none have adequately addressed effects of maternal or childhood geohelminths on asthma or atopy: (1) a birth cohort in Ethiopia that did not measure maternal geohelminths and in which the prevalence of geohelminths (<4%) in early childhood was too low to explore effects on allergy at 5 years^24^; and (2) a longitudinal study in Brazil, with no data on maternal geohelminths, showed that *Trichuris* infections in early childhood, particularly at higher parasite burdens, were associated with a reduced risk of SPT in later childhood.^25^ To our knowledge, the only other study to show effects of maternal geohelminths on allergy‐related outcomes was a study in Uganda that showed maternal hookworm reduced the risk of eczema in children.^26^

Previous cross‐sectional studies have shown that childhood geohelminths might protect against wheeze/asthma: (1) a study in Ethiopia in 1‐ to 4‐year‐olds showed a negative association between *Ascaris* and wheeze^27^; (2) a study among schoolchildren in a rural region in Ecuador showed an inverse association between heavy infections with *Trichuris* and atopic wheeze^28^—most previous cross‐sectional studies, however, showed no effects of *Trichuris* on asthma symptoms^9^, ^22^, ^29^, ^30^; and (3) three separate studies in Ethiopia showed an inverse relationship between hookworm infection and asthma symptoms.^9^ With respect to *Ascaris* in school‐age children, several studies have shown a positive association between infection or allergic sensitization to *Ascaris* antigens and asthma symptoms^9^, ^29^, ^31^, ^32^ and airways reactivity,^30^, ^32^, ^33^ an effect that was strongest in non‐atopics.^29^ Our data showed positive associations between greater parasite burdens with *Ascaris* in mothers and risk of asthma (Figure 4) and markers of airways inflammation (Table S9) in non‐atopic children, while *Ascaris* in children was associated with elevated FeNO (Table S9).

Our observation that maternal infections protect against atopy (Figure 2) is consistent with observations of inverse associations between geohelminths and SPT from cross‐sectional studies of schoolchildren.^22^, ^25^, ^34^ A protective effect of maternal geohelminth (against mite) was present from 3 years of age.^16^, ^17^ Childhood infections protected against SPT to perennial allergens from 5 years,^17^ and strongest effects at 8 years on SPT were observed among infected children of infected mothers. Maternal geohelminths were strongly associated with childhood infections to 5 years of age—reflecting a shared risk of infection in the household environment—a child growing up in a household where one or more family members are infected is at greater risk of infection.^35^ The previous observation from Brazil showing a protective effect of early‐life *Trichuris* infections against SPT at school age^25^ could have been mediated partly by maternal infections which were not measured but with which early childhood infections are likely to be strongly associated. A maternally mediated effect on SPT could explain two previous observations from Ecuador: (1) bimonthly anthelmintic treatments in schoolchildren showed no treatment effect on allergen SPT,^11^ and (2) community mass drug administrations with the broad‐spectrum anthelmintic, ivermectin, over 15 years for the elimination of onchocerciasis, were associated with an increase in SPT prevalence in schoolchildren.^36^ Long‐term ivermectin started before most children were born, likely resulted in reduced geohelminth infections in mothers.^36^

We have shown previously in this population that newborns of mothers infected with *Ascaris* have evidence of sensitization of CD4+ T cells to *Ascaris* antigens.^37^ The same is likely to be true for *T. trichiura* that has an intimate relationship with the mucosal immune system.^8^ Certainly, geohelminth antigens are present in the blood^38^ of infected mothers and can cross the placenta to sensitize the foetus. Decreased responsiveness could be associated with tolerization to parasite allergens including those that are cross‐reactive with aeroallergens. Extensive cross‐reactivity has been demonstrated between helminth parasites and aeroallergens,^39^ and such cross‐reactivity can mediate cross‐sensitization in immediate hypersensitivity skin reactions in murine models.^40^ The suppressive effect of maternal geohelminths on SPT (Figure 2) in children could occur through tolerization to cross‐reactive allergens.

Differences in the life cycle of the two principal geohelminth species present in the study setting could explain parasite species‐specific effects among children acquiring infections during childhood. *Trichuris* is exclusively enteric and has an intimate relationship with the host mucosa—it inserts its anterior end into the mucosa where it feeds—and has strong regulatory effects on mucosal inflammatory responses.^8^ Such an effect could explain the modulatory effect of early‐life trichuriasis on wheeze symptoms (Figure 3). In contrast, *Ascaris* has a phase of larval migration through the lungs where it can induce strong inflammatory responses.^8^ Childhood infections with *Ascaris* might be expected to increase eosinophilic inflammation in the airways and might explain elevated FeNO (Table S9). The transmission of maternal geohelminth effects on increasing airways symptoms, reactivity and inflammation to non‐atopic offspring is less clear. There is evidence from experimental models that the maternal immune response to a helminth infection may affect the risk of airways inflammation in offspring through effects on the fetomaternal interface^41^: maternal helminth infections in humans have been associated with increased pro‐inflammatory gene expression profiles in mother, placenta and foetus.^42^, ^43^ Such effects could lead to potentiated inflammatory responses in the airways of offspring. Interestingly, a maternal effect of *Trichuris* on increased wheeze in children was observed only among children who did not acquire *Trichuris* infections during childhood (Table S7), indicating that *in utero* effects could be modified by childhood infections.

Strengths of the study include prospective design with follow‐up from birth, stool data on maternal geohelminths during pregnancy and collection of large number of sociodemographic and lifestyle variables allowing us to control for potential confounders. Potential biases were reduced by using objective measures of geohelminth infections, performing all evaluations blind to the child's exposure status, and high retention in the cohort to 8 years (~80%). Repeated exposure measures for childhood geohelminths during the first 5 years of life provided more precise estimates of infection rates but children with positive stools were treated thus reducing prevalence and parasite burdens. SPT is a more reliable measure of atopy than allergen‐specific IgE in populations endemic for helminth parasites because of high proportions of false‐positive reactions in serologic assays caused by cross‐reactive carbohydrate determinants such as glycans.^44^, ^45^ We did exploratory analyses relating to effects of geohelminth parasite species and burden on outcomes and effects of exposures on airways reactivity and inflammation for which power was limited. Such findings should be interpreted with caution and require replication in future studies. Our definition of recent wheeze has been used widely in epidemiological studies and validated in different settings. It has the advantage of being readily understood in most language and cultural settings and may be less subject to bias in populations with limited access to health care. There is no widely agreed definition for asthma—the definition used here was designed to be more specific than recent wheeze but likewise may be subject to misclassification.

## 5 CONCLUSIONS

Evidence of a protective effect of STH parasites against allergy in children remains fragmentary and inconsistent. Our data indicate that maternal geohelminths protect children from the development of allergen SPT but increase the risk of wheeze, and airways reactivity and inflammation. The latter effects were attributable to specific parasite species. Early childhood *Trichuris* appeared to protect against wheeze. Overall, our findings indicate that *in utero* exposures to geohelminths through maternal infections may have long‐lasting effects on allergic inflammation and airways disease. These effects extended to school age and were modified by childhood infections, parasite species and atopy.

## CONFLICT OF INTEREST

None of the authors had any conflict of interest.

## AUTHOR CONTRIBUTIONS

Design – PJC, MLB, TAEPM, DPS; data collection – MEC, MV, YO, AM, PJC; Analysis ‐ PJC, ICS; interpretation – PJC, DPS, MLB, TAEPM; drafting manuscript – PJC; reviewing of manuscript and final approval – all authors.

## Supporting information

Table S1‐S9
