# Supplementary material for: Impact of early life geohelminths on wheeze, asthma and atopy in Ecuadorian children at 8 years
Source: Allergy. 2021 Apr 7;76(9):2765–75. doi: 10.1111/all.14821 (PMC8496980; doi:10.1111/all.14821)
Supplement: Supplementary file 1 — Table S1‐S9 [file ALL-76-2765-s001.docx]

Supplementary Tables

| Variable | Included  (n=1,933) | Excluded  (n=471) | P value |
| --- | --- | --- | --- |
| Maternal age (yrs)  ≤20  21-29  ≥30 | 25.9%  48.1%  26.0% | 30.4%  49.3%  20.4% | **0.021** |
| Maternal ethnicity  Non-Afro-Ecuadorian  Afro-Ecuadorian | 26.3%  73.7% | 22.9%  77.1% | 0.135 |
| Maternal educational level  Illiterate  Complete primary  Complete Secondary | 15.2%  58.6%  26.2% | 15.9%  59.2%  24.8% | 0.798 |
| Area of residence  Urban  Rural | 69.6%  30.4% | 72%  28% | 0.320 |
| Sex  Male  Female | 50.9%  49.1% | 51.8%  48.2% | 0.726 |
| Socioeconomic status§  1  2  3 | 33.2%  33.0%  33.8% | 32.9%  34.4%  32.7% | 0.834 |
| Birth order  1^st^  2^nd^-4^th^  ≥5^th^ | 25.4%  55.0%  10.6% | 23.4%  59.0%  17.6% | 0.283 |
| Maternal allergy  No  Yes | 95.4%  4.6% | 93.6%  6.4% | 0.117 |
| Household overcrowding¶  ≤3  >3 | 56.2%  43.8% | 54.4%  45.6% | 0.473 |
| Pets inside house  No  Yes | 74.4%  25.6% | 74.5%  25.5% | 0.892 |
| Large farm animals‡  No  Yes | 66.9%  33.1% | 68.6%  31.4% | 0.484 |
| Pneumonia to 13 months  No  Yes | 95.3%  4.7% | 96.1%  3.9% | 0.417 |

Table 1. Comparison of frequencies of potential confounders between cohort children included or excluded from the analysis.

P<0.05 are shown in bold. §Socioeconomic status (SES) represents tertiles of z scores obtained using a factor analysis with 1 representing the lowest and 3 the highest SES. ¶ Household overcrowding is defined as the number of people living in the household per sleeping room. ‡ Any of cows, pigs, mules, donkeys, and horses.

| Variable | Overall | Wheeze | | | Asthma | | | SPT to any allergen | | |
| --- | --- | --- | --- | --- | --- | --- | --- | --- | --- | --- |
|  | n (%) | % | OR (95% CI) | P value | % | OR (95% CI) | P value | % | OR (95% CI) | P value |
| Childhood geohelminths to 13 m  Any geohelminth  No  Yes  *Ascaris*  No  Yes  *Trichuris*  No  Yes  Childhood geohelminths to 2 yr  Any geohelminth  No  Yes  *Ascaris*  No  Yes  *Trichuris*  No  Yes  Childhood geohelminths to 3 yr  Any geohelminth  No  Yes  *Ascaris*  No  Yes  *Trichuris*  No  Yes | 1713 (90.9%)  172 (9.1%)  1760 (92.8%)  136 (7.2%)  1876 (97.8%)  45 (2.3%)  1342 (71.2%)  543 (28.8%)  1471 (77.9%)  417 (22.1%)  1650 (87.4%)  238 (12.6%)  1169 (61.5%)  733 (38.5%)  1318 (69.3%)  584 (30.7%)  1545 (81.2%)  357 (18.8%) | 6.8  3.5  6.7  4.4  6.7  0  7.1  5.4  6.7  6.2  7.0  4.2  6.0  6.0  6.8  6.2  7.1  4.5 | 1  0.49 (0.21-1.14)  1  0.65 (0.28-1.51)  -----  1  0.75 (0.48-1.15)  1  0.94 (0.60-1.48)  1  0.57 (0.29-1.11)  1  0.83 (0.56-1.24)  1  1.01 (0.66-1.53)  1  0.57 (0.32-1.00) | 0.098  0.313  0.187  0.783  0.096  0.370  0.958  0.052 | 7.9  7.6  7.8  9.6  8.1  2.2  8.1  7.1  7.8  7.9  8.1  6.3  8.4  6.8  8.4  7.2  8.2  5.9 | 1  0.86 (0.47-1.58)  1  1.17 (0.64-2.15)  1  0.22 (0.03-1.64)  1  0.77 (0.52-1.14)  1  0.94 (0.62-1.42)  1  0.66 (0.37-1.15)  1  0.72 (0.49-1.04)  1  0.92 (0.61-1.36)  1  0.62 (0.37-1.03) | 0.637  0.607  0.140  0.186  0.763  0.144  0.082  0.664  0.066 | 15.4  9.9  15.1  11.8  14.9  6.7  15.7  12.6  15.6  12.0  15.0  13.5  16.2  12.3  15.7  12.5  15.2  12.6 | 1  0.61 (0.36-1.03)  1  0.76 (0.44-1.31)  1  0.41 (0.13-1.35)  1  0.79 (0.59-1.07)  1  0.77 (0.55-1.07)  1  0.90 (0.60-1.34)  1  0.77 (0.58-1.03)  1  0.86 (0.63-1.17)  1  0.90 (0.62-1.32) | 0.067  0.325  0.143  0.127  0.115  0.601  0.078  0.334  0.605 |

Table 2. Adjusted associations between childhood geohelminths and parasite type to 13 months, and 2 and 3 years of age, and wheeze and allergen skin test (SPT) reactivity to any allergen at 8 years and asthma between 5 and 8 years

SPT – allergen skin prick test reactivity to any of 9 allergens. Odds ratios (OR) and 95% confidence intervals (95% CI) were estimated using logistic regression. P<0.05 are shown in bold. m – months; yr – years. Analyses were adjusted for maternal geohelminths, childhood geohelminth parasite type, maternal ethnicity and allergy, area of residence, sex, birth order, and large animals around the household.

| Variable | Overall | Wheeze | | | Asthma | | | SPT to any allergen | | |
| --- | --- | --- | --- | --- | --- | --- | --- | --- | --- | --- |
|  | n (%) | % | OR (95% CI) | P value | % | OR (95% CI) | P value | % | OR (95% CI) | P value |
| Maternal geohelminth  *Ascaris*  No  Yes  Intensity  Uninfected  Light  Moderate/heavy  *Trichuris*  No  Yes  Intensity  Uninfected  Light  Moderate/heavy  Hookworm  No  Yes | 1400 (72.4)  533 (27.6)  1485 (77.5)  330 (17.2)  101 (5.3)  1374 (71.1)  559 (28.9)  1420 (74.1)  419 (21.9)  77 (4.0)  1824 (94.4)  109 (5.6) | 6.2  7.0  6.5  6.7  6.9  6.2  7.5  6.2  7.6  7.8  6.6  6.4 | 1  0.89 (0.58-1.38)  1  0.92 (0.55-1.53)  1.02 (0.44-2.36)  1  1.25 (0.82-1.89)  1  1.17 (0.39-3.46)  1.23 (0.33-4.68)  1  1.01 (0.45-2.28) | 0.612  0.745  0.965  0.291  0.781  0.758  0.974 | 8.2  7.6  7.6  8.2  11.9  7.9  7.9  7.8  8.1  9.1  8.2  2.8 | 1  1.00 (0.68-1.49)  1  1.08 (0.68-1.71)  1.77 (0.89-3.53)  1  0.93 (0.63-1.39)  1  1.52 (0.44-5.17)  1.77 (0.43-7.33)  1  0.34 (0.11-1.12) | 0.987  0.744  0.103  0.734  0.507  0.433  0.076 | 16.8  12.2  15.6  11.5  12.9  16.0  11.5  16.1  11.7  7.8  14.9  11.0 | 1  0.78 (0.57-1.08)  1  0.78 (0.53-1.14)  1.03 (0.55-1.93)  1  0.75 (0.55-1.03)  1  0.75 (0.35-1.63)  0.51 (0.17-1.53)  1  0.86 (0.46-1.62) | 0.134  0.201  0.929  0.077  0.467  0.226  0.645 |
| Child geohelminths to 5 yr  *Ascaris*  No  Yes  *Trichuris*  No  Yes | 1223 (63.7)  697 (36.3)  1430 (74.5)  490 (25.5) | 6.5  6.7  7.1  4.9 | 1  1.22 (0.81-1.84)  1  **0.57 (0.35-0.94)** | 0.331  **0.029** | 7.8  7.8  8.3  6.3 | 1  1.05 (0.72-1.54)  1  0.64 (0.41-1.01) | 0.793  0.056 | 15.9  12.6  15.2  13.1 | 1  0.86 (0.64-1.16)  0.95 (0.68-1.32) | 0.320  0.750 |
| Child geohelminths at 5 yr  Any  No  Yes  *Ascaris*  No  Yes  Intensity  Uninfected  Light  Moderate/heavy  *Trichuris*  No  Yes  Intensity  Uninfected  Light  Moderate/heavy | 1197 (74.8)  404 (25.2)  1358 (84.8)  243 (15.2)  1376 (86.1)  162 (10.1)  60 (3.8)  1335 (83.5)  264 (16.5)  1364 (85.4)  202 (12.6)  32 (2.0) | 6.9  5.9  6.3  8.2  6.4  6.2  11.4  6.9  5.3  6.7  5.5  9.4 | 1  0.80 (0.49-1.32)  1  1.60 (0.92-2.78)  1  1.15 (0.57-2.32)  **2.83 (1.13-7.13)**  1  0.57 (0.30-1.07)  1  0.71 (0.35-1.41)  1.04 (0.29-3.76) | 0.389  0.099  0.705  **0.027**  0.082  0.325  0.954 | 7.9  8.2  7.7  9.5  7.6  6.8  15.0  7.9  8.0  7.6  8.4  9.4 | 1  0.98 (0.63-1.52)  1  1.31 (0.78-2.22)  1  0.91 (0.47-1.79)  **2.60 (1.13-6.00)**  1  0.81 (0.47-1.39)  1  0.99 (0.55-1.77)  0.97 (0.28-3.49) | 0.928  0.298  0.790  **0.025**  0.443  0.908  0.965 | 14.5  14.1  14.5  13.6  14.6  16.1  8.3  14.5  13.6  14.5  13.4  15.6 | 1  1.10 (0.78-1.55)  1  1.04 (0.67-1.60)  1  1.22 (0.77-1.96)  0.64 (0.24-1.68)  1  1.00 (0.65-1.52)  1  0.99 (0.62-1.57)  1.21 (0.44-3.45) | 0.579  0.870  0.398  0.361  0.992  0.955  0.714 |

Table 3. Adjusted analyses for associations between geohelminth parasite species and parasite burdens in mothers and in children up to and at 5 years of age, and wheeze and allergen skin test (SPT) reactivity to any allergen at 8 years and asthma between 5 and 8 years

SPT – allergen skin prick test reactivity to any of 9 allergens. Odds ratios (OR) and 95% confidence intervals (95% CI) were estimated using logistic regression and adjusted for maternal allergy and ethnicity, area of residence, sex, birth order, contact with large farm animals, geohelminths in the child to 5 years (for maternal geohelminths), maternal geohelminths (for childhood geohelminths), and presence of other species of geohelminth parasite. Infection intensity categories are: *Ascaris*, light (<5,000 epg), moderate/heavy (>5,000 epg); *Trichuris*, light (<1000 epg), moderate/heavy (>1,000 epg). P<0.05 are shown in bold. Denominators for geohelminth prevalence versus intensity results differ because it was not possible to do Kato-Katz examinations on all samples where sample volume was insufficient. P<0.05 are shown in bold.

| Category | n | % | OR (95% CI) | P value |
| --- | --- | --- | --- | --- |
| Mother-/child- | 681 | 17.3 | 1 |  |
| Mother+/child- | 358 | 14.3 | 0.77 (0.54-1.11) | 0.159 |
| Mother-/child+ | 361 | 15.5 | 0.88 (0,62-1.26) | 0.492 |
| Mother+/child+ | 520 | 11.0 | **0.58 (0.41-0.83)** | **0.003** |

Table 4. Adjusted associations for 4-way analysis of strata of presence/absence of maternal and childhood geohelminths and allergen skin prick test (SPT) reactivity to any allergen at 8 years.

SPT – allergen skin prick test reactivity to any of 9 allergens. Odds ratios (OR) and 95% confidence intervals (95% CI) were estimated using logistic regression. P<0.05 are shown in bold. Analyses were adjusted maternal ethnicity and allergy, area of residence, sex, birth order, and large animals around the household.

| Variable | Airways reactivity | | | FeNO (>35 ppb) | | | Nasal eosinophilia (>5%) | | |
| --- | --- | --- | --- | --- | --- | --- | --- | --- | --- |
|  | % | Adjusted  OR (95% CI) | P value | % | Adjusted  OR (95% CI) | P value | % | Adjusted  OR (95% CI) | P value |
| Any maternal geohelminths  No  Yes | 9.4  10.7 | 1  1.01 (0.77-1.31) | 0.654 | 10.1  10.5 | 1  0.92 (0.67-1.25) | 0.581 | 9.3  9.1 | 1  0.95 (0.64-1.38) | 0.777 |
| Any childhood geohelminths  No  Yes | 9.1  11.1 | 1  1.04 (0.80-1.35) | 0.826 | 9.3  11.5 | 1  1.24 (0.91-1.70) | 0.177 | 9.7  8.6 | 1  0.88 (0.60-1.30) | 0.529 |

Table 5. Adjusted associations between maternal and childhood geohelminths (to 5 years) and airways reactivity and elevated markers of airways inflammation including fractional exhaled nitric oxide (FeNO) and nasal eosinophilia (>5%).

Samples available for analysis were airways reactivity (n=1872). FeNO (n=1887) and nasal eosinophilia (n=1412). ppb – parts per billion. Odds ratios (OR) and 95% confidence intervals (95% CI) were estimated using logistic regression. P<0.05 are shown in bold. Associations were adjusted for maternal/childhood geohelminths, maternal ethnicity and allergy, area of residence, sex, birth order, and large animals around the household

| Variable | Wheeze | | | | | Asthma | | | | |
| --- | --- | --- | --- | --- | --- | --- | --- | --- | --- | --- |
|  | SPT- | | SPT+ | | Interact. P value | SPT- | | SPT+ | | Interact. P value |
|  | OR (95% CI) | P value | OR (95% CI) | P value |  | OR (95% CI) | P value | OR (95% CI) | P value |  |
| Maternal geohelminths  Any  No  Yes  *Ascaris*  No  Yes  Intensity  Uninfected  Light  Moderate/heavy  *Trichuris*  No  Yes  Intensity  Uninfected  Light  Moderate/heavy | 1  **1.73 (1.06-2.83)**  1  1.12 (0.67-1.88)  1  1.07 (0.58-1.99)  1.61 (0.67-3.87)  1  **1.78 (1.08-2.93)**  1  **1.74 (1.01-2.97)**  2.25 (0.87-5.78) | **0.028**  0.657  0.824  0.283  **0.024**  **0.044**  0.093 | 1  0.62 (0.31-1.29)  1  0.68 (0.28-1.63)  1  0.87 (0.32-22.37)  -------  1  0.74 (0.31-1.73)  1  0.81 (0.33-2.00)  ------ | 0.206  0.387  0.785  ------  0.482  0.655  ------- | **0.029**  0.182  0.557  0.058  0.159 | 1  1.06 (0.70-1.59)  1  1.14 (0.73-1.77)  1   - 1. 0.71-2.01)   **2.11 (1.01-4.38)**  1  1.01 (0.65-1.59)  1  1.10 (0.68-1.79)  1.14 (0.46-2.86) | 0.796  0.564  0.498  **0.046**  0.949  0.681  0.776 | 1  **0.40 (0.18-0.92)**  1  0.76 (0.30-1.92)  1  0.94 (0.32-2.77)  0.85 (0.09-7.97)  1  0.51 (0.19-1.36)  1  0.40 (0.13-1.270  1.17 (0.11-12.43) | **0.031**  0.560  0.910  0.890  0.177  0.120  0.894 | **0.030**  0.267  0.595  0.150  0.084  **0.048**  0.675 |
| Child geohelminths to 5 yr  Any  No  Yes  *Ascaris*  No  Yes  *Trichuris*  No  Yes | 1  0.89 (0.55-1.45)  1  1.24 (0.75-2.06)  1  0.57 (0.30-1.03) | 0.647  0.405  0.062 | 1  1.00 (0.50-2.01)  1  1.37 (0.64-2.91)  1  0.52 (0.21-1.31) | 0.995  0.420  0.164 | 0.924  0.747  0.592 | 1  0.85 (0.56-1.29)  1  1.21 (0.78-1.86)  1  0.60 (0.36-1.00) | 0.442  0.390  0.051 | 1  0.73 (0.34-1.59)  1  0.72 (0.30-1.71)  1  0.69 (0.26-1.84) | 0.431  0.452  0.461 | 0.582  0.123  0.724 |

Table 6. Adjusted analyses for associations between geohelminths, geohelminth parasite species and parasite burdens in mothers and children and wheeze/asthma in later childhood, stratified by presence (+) and absence (-) of allergen skin prick test reactivity.

SPT – allergen skin prick test reactivity to any of 9 allergens (SPT+, n=1649; SPT-, n=284). Odds ratios (OR) and 95% confidence intervals (95% CI) were estimated using logistic regression and adjusted for maternal allergy and ethnicity, area of residence, sex, birth order, contact with large farm animals, geohelminths in the child to 5 years (for maternal geohelminths), maternal geohelminths (for childhood geohelminths), and presence of other species of geohelminth parasite. P<0.05 are shown in bold. Interaction P values are shown for adjusted models but did not differ markedly from those of unadjusted models. Infection intensity categories are: *Ascaris*, light (<5,000 epg), moderate/heavy (>5,000 epg); *Trichuris*, light (<1000 epg), moderate/heavy (>1,000 epg). Denominators for geohelminth prevalence versus intensity results differ because it was not possible to do Kato-Katz examinations on all samples where sample volume was insufficient. P<0.05 are shown in bold.

| Category | n | % | OR (95% CI) | P value |
| --- | --- | --- | --- | --- |
| Mother-/child- | 933 | 3.8 | 1 |  |
| Mother+/child- | 279 | 9.0 | **2.39 (1.39-4.10)** | **0.002** |
| Mother-/child+ | 215 | 3.7 | 0.94 (0.43-2.08) | 0.878 |
| Mother+/child+ | 211 | 3.8 | 0.93 (0.42-2.07) | 0.858 |

Table 7. Adjusted associations for 4-way analysis of strata of presence/absence of maternal and childhood trichuriasis and wheeze at 8 years among non-atopic children

Odds ratios (OR) and 95% confidence intervals (95% CI) were estimated using logistic regression. P<0.05 are shown in bold. Analyses were adjusted maternal ethnicity and allergy, area of residence, sex, birth order, and large animals around the household.

| Variable | SPT- | | | | SPT+ | | | |
| --- | --- | --- | --- | --- | --- | --- | --- | --- |
|  | N | n(%) | OR (95% CI) | P value | N | n(%) | OR (95% CI) | P value |
| Airways reactivity  Maternal geohelminths  No  Yes  Childhood geohelminths  No  Yes | 850  749  861  738 | 79 (9.3)  79 (10.6)  78 (9.1)  80 (10.8) | 1  1.06 (0.80-1.40)  1  0.98 (0.74-1.30) | 0.696  0.890 | 170  105  163  112 | 17 (10.0)  105 (11.4)  15 (9.2)  14 (12.5) | 1  0.67 (0.31-1.46)  1  1.67 (0.79-3.51) | 0.312  0.180 |
| Elevated FeNO  Maternal geohelminths  No  Yes  Childhood geohelminths  No  Yes | 851  758  861  748 | 63 (7.4)  60 (7.9)  53 (6.2)  70 (9.4) | 1  0.90 (0.61-1.33)  1  1.46 (0.99-2.16) | 0.605  0.059 | 173  105  165  113 | 40 (23.1)  31 (29.5)  42 (25.5)  29 (25.6) | 1  1.26 (0.69-2.28)  1  0.98 (0.54-1.80) | 0.449  0.950 |
| Nasal eosinophilia  Maternal geohelminths  No  Yes  Childhood geohelminths  No  Yes | 631  575  648  558 | 41 (6.5)  46 (8.0)  44 (6.8)  43 (7.7) | 1  1.17 (0.74-1.85)  1  1.09 (0.69-1.72) | 0.496  0.715 | 130  76  126  80 | 30 (23.1)  13 (17.1)  31 (24.6)  12 (15.0) | 1  0.66 (0.29-1.48)  1  0.49 (0.22-1.09) | 0.314  0.081 |

Table 8. Adjusted associations between maternal and childhood geohelminth infections to 5 years and airways reactivity and elevated markers of airways inflammation including fractional exhaled nitric oxide (FeNO) and nasal eosinophilia (>5%), stratified by stratified by presence (+) and absence (-) of atopy measured by allergen skin prick test (SPT) reactivity

SPT – allergen skin prick test reactivity to any of 9 allergens. Odds ratios (OR) and 95% confidence intervals (95% CI) were estimated using logistic regression. P<0.05 are shown in bold. Elevated FeNO (fractional exhaled nitric oxide >35 parts per billion). Nasal eosinophilia (>5%). Reversibility – increase in FEV1 of 12% or more post-bronchodilator. Sample for analysis was FeNO (n=1887), nasal eosinophilia (n=1412), and airways reactivity (n=1872). Analyses were adjusted for maternal/childhood geohelminths, maternal ethnicity and allergy, area of residence, sex, birth order, and large animals around the household.

| Variable | Airways reactivity | | | FeNO | | | Nasal eosinophilia | | |
| --- | --- | --- | --- | --- | --- | --- | --- | --- | --- |
|  | % | OR (95% CI) | P value | % | OR (95% CI) | P value | % | OR (95% CI) | P value |
| Maternal geohelminth  *Ascaris*  No  Yes  Intensity  Uninfected  Light  Moderate/heavy  *Trichuris*  No  Yes  Intensity  Uninfected  Light  Moderate/heavy | 9.5  10.8  9.6  11.0  10.8  8.7  12.7  8.6  13.5  14.5 | 1  0.94 (0.64-1.38)  1  0.92 (0.59-1.44)  0.80 (0.37-1.72)  1  1.42 (0.98-2.03)  1  **1.56 (1.05-2.01)**  1.68 (0.80-3.55) | 0.745  0.709  0.574  0.066  **0.028**  0.173 | 7.2  8.7  7.0  7.3  19.5  7.5  8.1  7.6  8.0  8.7 | 1  1.09 (0.72-1.66)  1  0.97 (0.58-1.65)  **2.89 (1.53-5.49)**  1  0.91 (0.60-1.40)  1  0.90 (0.57-1.44)  0.90 (0.36-2.21) | 0.680  0.923  **0.001**  0.674  0.664  0.811 | 6.5  9.1  6.7  7.4  14.5  6.9  7.9  6.7  8.2  9.1 | 1  1.38 (0.85-2.24)  1  1.05 (0.58-2.91)  **2.27 (1.00-5.12)**  1  1.01 (0.62-1.66)  1  1.10 (0.65-1.86)  1.09 (0.40-2.98) | 0.188  0.861  **0.049**  0.957  0.726  0.869 |
| Child geohelminths to 5 yr  *Ascaris*  No  Yes  *Trichuris*  No  Yes | 8.7  11.7  10.2  8.8 | 1  1.40 (0.97-2.02)  1  **0.62 (0.40-0.96)** | 0.069  **0.031** | 6.2  10.2  7.2  9.0 | 1  **1.61 (1.07-2.42)**  1  0.99 (0.63-1.56) | **0.021**  0.963 | 6.9  7.9  7.2  7.3 | 1  1.14 (0.71-1.85)  1  0.88 (0.51-1.53) | 0.590  0.660 |

Table 9. Adjusted associations between geohelminth infections in mothers and in children up to and at 5 years and and airways reactivity, and elevated markers of airways inflammation including fractional exhaled nitric oxide (FeNO) and nasal eosinophilia (>5%) among non-atopic children.

Samples available for analysis were airways reactivity (n=1,599), FeNO (n=1,609) and nasal eosinophilia (n=1,206). ppb – parts per billion. Odds ratios (OR) and 95% confidence intervals (95% CI) were estimated using logistic regression. P<0.05 are shown in bold. Analyses were adjusted for maternal geohelminths, childhood geohelminth parasite type, maternal ethnicity and allergy, area of residence, sex, birth order, and large animals around the household. yr – years
